# A Printable OECT for Simple Integration in Nitrocellulose-Based Assays

**DOI:** 10.1021/acssensors.5c01893

**Published:** 2025-09-30

**Authors:** Martina Cicolini, Ali Solgi, Lorenzo Vigna, Alberto Ballesio, Simone Marasso, Matteo Cocuzza, Hans Kleemann, Francesca Frascella, Lucia Napione

**Affiliations:** † Department of Applied Science and Technology (DISAT), 19032 Politecnico di Torino, Corso Duca Degli Abruzzi 24, 10129 Turin, Italy; ‡ PolitoBioMed Lab, Department of Applied Science and Technology (DISAT), Politecnico di Torino, Corso Duca Degli Abruzzi 24, 10129 Turin, Italy; § PiQuET LAB, Piemonte Quantum Enabling Technology, Str. delle Cacce, 91, 10135 Turin, Italy; ∥ Institute of Materials for Electronics and Magnetism, IMEM-CNR, Parco Area delle Scienze 37/A, 43124 Parma, Italy; ⊥ Dresden Integrated Center for Applied Physics and Photonic Materials (IAPP), 9169 Technische Universität Dresden, Nöthnitzer Str. 61, 01187 Dresden, Germany

**Keywords:** 3D printed electronics, organic semiconductors, OECT, additive manufacturing, paper-based sensors, PEDOT:PSS

## Abstract

Paper-based biosensors hold significant promise for point-of-care (POC) diagnostic applications. Among these, lateral flow assays (LFAs) are particularly appealing due to their ease of use, portability, and low cost. However, their limited sensitivity and qualitative output set drawbacks on their reliability and widespread application. In response to the growing need for rapid and consistent diagnostic and monitoring tools, Organic Electrochemical Transistors (OECTs) have emerged as powerful devices in biochemical sensing applications because of their high sensitivity, low operating voltage, and compatibility with a biological environment. In this work, we developed a printable OECT for biochemical sensing on a commercial cellulose membrane, commonly used as a detection substrate in LFA-based rapid tests. Constituting a self-standing, passive microfluidic platform, the system was designed to transport and interact with liquid samples, while ensuring a contamination-free zone for the active components. Inside a dry area delimited by a hydrophobic barrier, the OECT components include dispense-printed silver electrodes, a polystyrenesulfonate-doped poly­(3,4-ethylenedioxy-thiophene) (PEDOT:PSS) channel and gate, and a solid-state electrolyte (SSE) layer. Outside the dry area, a PEDOT:PSS extended gate alone interacts with the analyte in the liquid sample, preventing channel contamination and enhancing the system stability. We investigated the effect of dopamine (DA) oxidation at the extended gate interface on the device response and observed variations in the transfer characteristics, transconductance and *I*_on_/*I*_off_ ratio, obtaining a limit of detection of 0.01 mM. With a maximum transconductance of approximately 4 mS, our system shows potential for the integration of an easy-to-fabricate device into an affordable biochemical assay, providing quantitative results at the point-of-care site to complement and reinforce the typical colorimetric response of LFAs.

## Graphical abstract

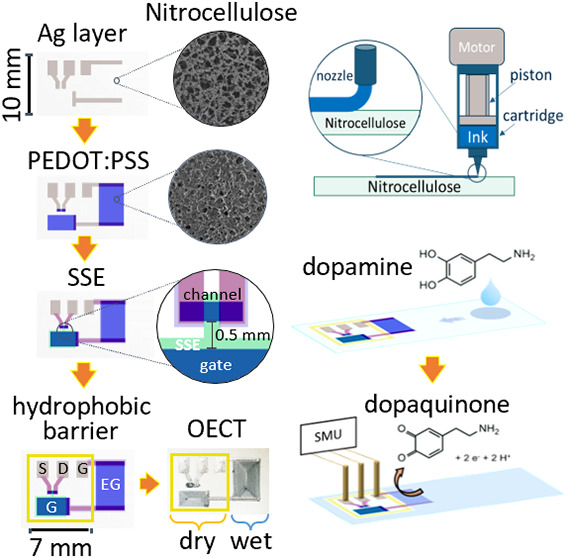

In an increasingly globalized and interconnected world, mass testing represents a crucial strategy to minimize disease spread—not only within healthcare but also in the food safety and agriculture sectors.^1^ The COVID-19 pandemic drew attention to how reliance on centralized laboratory testing can be a limitation in emergency situations, both in terms of time and cost. The growing interest in portable sensing and monitoring devices stems from the need for reliable diagnostic tests at the point-of-care (POC) site—defined as the location of the patient or sampling point—capable of reducing the burden on hospitals and clinical analysis laboratories.^2,3^

In this context, lightweight and affordability represent key features for the materials selection in the development of effective POC tools. Among the ones meeting these requirements, cellulose has been drawing interest as an appealing substrate for POC biosensors, also due to its abundance and renewability.^4^ Lateral flow assays (LFAs) are well established and commercially available cellulose-based biochemical sensors, which gained particular attention during the COVID-19 pandemic for their use as rapid tests in decentralized POC screenings. Based on overlapping cellulose pads, LFAs are driven by capillarity, constituting self-standing microfluidic platforms. The detection zone is composed of nitrocellulose, an amine-rich paper commonly employed in biochemistry for its ability to immobilize biomolecules. The colorimetric output signal of LFAs is given by the aggregation of metal nanoparticles upon the occurrence of antibody–antigen bindings at the control and test lines.^5^

Despite their matchless simplicity of use and their outstanding potential in point-of-care testing, LFAs are also characterized by poor sensitivity, together with the subjective interpretation of the colorimetric signal.^6,7^ To address these issues, many studies have been focusing on either amplifying LFAs colorimetric signal^8–10^ or on integrating the system with an analytical component, able to provide an independent output from the colorimetric one.^11–13^ This solution usually involves the use of screen-printed electrochemical sensors, whose operation requires the addition of a redox mediator to the assay, like ferrocene, and are typically suited for detecting higher concentration ranges.^14^

Recently, organic transistors have attracted interest in many different application fields as miniaturized, highly sensitive analytical devices. Organic electrochemical transistors (OECTs) are specifically suitable for biosensing applications, thanks to their low operational voltage and compatibility with the aqueous biological environment.^15–17^ Consisting of three electrodes immersed in an electrolyte, OECTs operate through the mixed ionic-electronic conduction occurring in the organic semiconductor (OSC) film connecting source and drain electrodes, mediated by the application of voltages at the gate electrode.^18^ OECTs can work as biosensors when the target in the electrolyte interferes with the electrochemical processes occurring in the system, such as ions or redox-active molecules like H_2_O_2_ ^19^ or dopamine,^20^ or when binding events, like antibody–antigen specific recognition, at the gate electrode surface modify its electrical properties, thereby shifting the output signal.^21,22^

Thanks to its biocompatible, water-based composition and high hole conductivity, PEDOT:PSS is considered a benchmark material for OSCs used in fabricating OECTs channels.^23^ Its tunable formulation and the extensive availability of products for solution processing on the market make additive manufacturing techniques widely adopted for the deposition of PEDOT:PSS thin films.^24–28^ Furthermore, the progressive refinement and diffusion of three-dimensional (3D) printing techniques, together with the higher attention over sustainable and high-throughput processes and materials, have been leading toward the exploration of innovative methods to be implemented in every step of OECTs manufacturing, to find simpler alternatives to the traditional fabrication techniques.^29–33^

Dispense printers are noncontact printers for high viscosity inks.^34^ The Voltera V-One PCB printer is a commercially available and cost-effective dispense printer that has recently been employed for the fabrication of printable antennas^34^, a gas sensor on paper^35^, and the rapid prototyping of OECTs on flexible polymeric substrates.^30^ However, to the best of our knowledge, nitrocellulose and LFA strips have not yet been explored as substrates for OECT fabrication, likely due to the inherent porosity and increased fragility of cellulose compared to conventional smooth substrates, which present challenges for device design and the use of standard manufacturing processes.

In this work, we developed a printable OECT on nitrocellulose, to enable easy and seamless integration of an analytical device into nitrocellulose-based biochemical assays. The chosen substrate acts as an independent microfluidic platform, conveniently enabling liquid transport during the operation of the device. A hydrophobic barrier, drawn using a permanent marker, encloses the PEDOT:PSS channel and gate to prevent contamination, creating a dry area inaccessible to the liquid sample. In this dry region, gate and channel communicate via the presence of a solid-state electrolyte (SSE) layer,^35^ composed of a polymeric matrix containing an ionic liquid; a PEDOT:PSS extended gate lies outside the barrier, allowing interactions with the analyte in the liquid vector (Figure 1b). This configuration ensures a clear separation between the sensing and active areas, not only allowing for easy future functionalization of the extended gate without risking contamination of other OECT components, but also preventing unspecific interactions of the analyte with the channel, so that any variation in the system response can be attributed solely to surface events occurring at the sensing extended gate. The dispense-printing technique well adapts to the delicate nitrocellulose substrate thanks to the highly customizable printing parameters, and it represents a highly cost-effective alternative to other expensive additive manufacturing techniques, like inkjet and aerosol jet printers, making it ideal for low-cost POC devices. Ultimately, our proposed strategy aims to integrate an OECT-based sensor with an LFA strip into an innovative system, providing users at the POC site with an analytical output that reinforces the typical colorimetric response of LFAs-based rapid tests.

**Figure 1:**
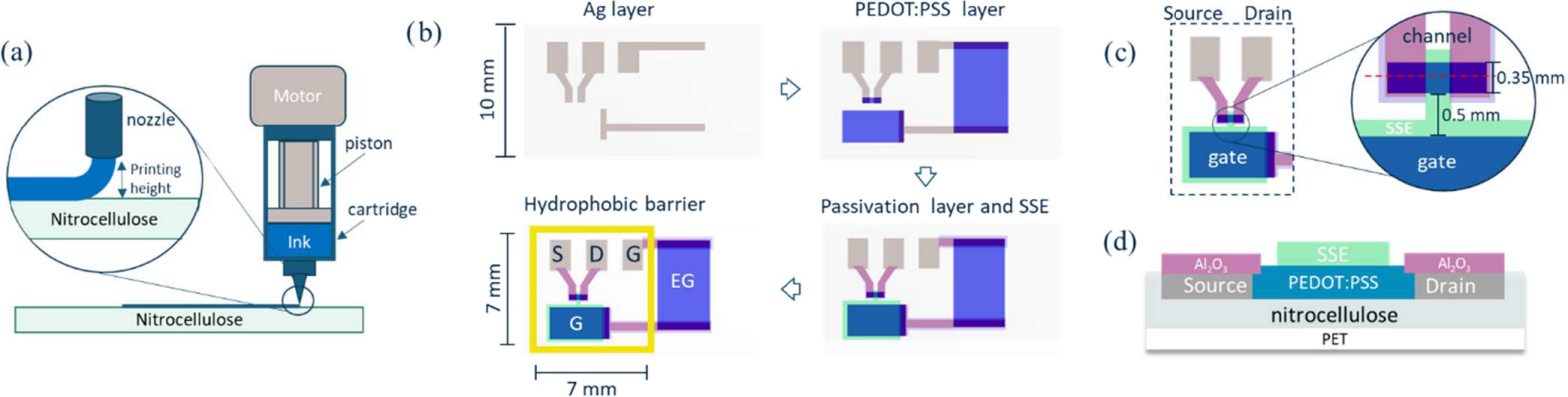
(a) Overview of the dispense-printer components and deposition process. (b) Schematics of the nitrocellulose-based OECT fabrication steps: silver (gray) electrode printing; PEDOT:PSS (blue) channel, gate and extended gate printing; Al_2_O_3_ (pink) and SSE (green) layer deposition; drawing of the hydrophobic barrier (yellow). The letters indicate source (S), drain (D), gate (G), and extended gate (EG). (c) Details of the OECT channel and its distance from the gate. The dashed red line indicates the cutting plane for the cross-sectional view shown in (d). (d) Schematics of the cross-section of the OECT channel.

## Experimental section

### Materials

The silver ink Voltera Conductor 3 was purchased from Ventaja Tecnológica. PEDOT:PSS Clevios SV4 was purchased from Heraeus.

Kapton sheets, Whatman FF170HP Din A nitrocellulose membrane sheets, *N*-isopropylacrylamide monomer, cross-linker *N*,*N*′-methylenebis­(acrylamide), photoinitiator 2-hydroxy-4′-(2-hydroxyethoxy)-2-methylpropiophenone, the ionic liquid 1-ethyl-3-methylimidazolium ethyl sulfate, dopamine hydrochloride, and all other chemicals were purchased from Sigma-Aldrich and were used as received, without further purification. Deionized water was obtained using a reverse osmosis (RO) purification system.

### Solid-State Electrolyte Preparation

As reported by Weissbach et al.,^35^ the SSE precursor was obtained by mixing 1.0 mL of deionized water, 750.0 mg of *N*-isopropylacrylamide, 20.0 mg of cross-linker, 200.0 mg of photoinitiator, and 1.5 mL of ionic liquid. The precursor was stirred overnight at room temperature.

### Device Fabrication

After designing the device layout using CleWin software, the silver electrodes and the PEDOT:PSS channel and gates were printed using the Voltera V-One PCB printer. Briefly, the silver electrodes for source, drain, gate and extended gate were printed on the nitrocellulose substrate using a disposable nozzle (internal diameter = 230 μm), and the ink was baked at 110 °C for 15 min. A Voltera cartridge was filled with PEDOT:PSS after mixing the paste, and a custom ink was created via the Voltera software to adapt the printing parameters to the properties of the PEDOT:PSS pastes. Three layers of PEDOT:PSS were deposited on top of the silver tracks using a 215 μm nozzle and implementing the “aligned printing” function, in order to fabricate the channel (channel length *L* = 170 μm, channel width *W* = 350 μm), gate (gate length *L* = 3500 μm, gate width *W* = 2100 μm) and extended gate (extended gate length *L* = 5500 μm, extended gate width *W* = 4000 μm), with a distance of 0.5 mm between the channel and gate (Figure 1c). After baking (110 °C for 30 min) and complete cooling, the silver electrodes were passivated via deposition of a 60 nm layer of Al_2_O_3_ by e-beam evaporation, using the E-gun evaporator Temescal FC-2000, through a custom laser-cut Kapton mask (power = 11%, frequency = 1500 Hz). At this point, 3 μL of SSE were drop casted and distributed on top of channel and gate inside the dry area, to create a thin layer. The SSE layer was cross-linked via UV exposure for 20 s. Lastly, a permanent marker was used to track a hydrophobic barrier around the channel and gate, so to create a dry area inaccessible to the liquid, thereby protecting the channel and gate from potential contamination by the sample.

### Electrical and Physical Characterization

Electrical measurements were performed using a Keysight source/measure unit (SMU) under ambient conditions. Three cycles of transfer characteristics were acquired at a fixed drain voltage of −0.4 V, sweeping the voltage applied to the extended gate (*V*_GS_) from −0.6 to 1.2 V, at a scan rate of 61.2 mV/s. Output characteristics were acquired at a gate voltage of −0.2 V, sweeping the drain voltage between 0 V and −0.6 V. The *I*_on_/*I*_off_ ratio and the transconductance of the OECTs were extracted from their transfer curves. *g*_m_ was normalized by dividing the values by the dimension factors *Wd*/*L*. The scanning electron microscopy (SEM) images were acquired with a FEI Quanta 3D FEG dual-beam (SEM/FIB) using an accelerating voltage of 5 kV. Nitrocellulose printed with PEDOT:PSS layers was not subjected to metallization, while a 50 nm Pt layer was sputtered on bare nitrocellulose to observe its morphology prior to any printing step.

## Results and Discussion

In this section, the design and characterization of the OECT on paper are described. The device was directly printed on a strip of commercial nitrocellulose membrane of approximately 1 × 1.5 cm^2^, having a standard thickness of about 200 μm (Figure 2a). Nitrocellulose is a gold standard material for the fabrication of rapid diagnostic tests, thanks to its inherent ability to retain biomolecules and its optimized porosity.^36^ In this work, nitrocellulose was chosen as substrate with the aim of integrating an analytical device into a passive microfluidic platform, developing a fast and low-cost fabrication procedure for OECTs. The details of the cost estimate for materials and fabrication can be found in Tables S1 and S2.

The Voltera V-One dispensing printer enabled high-throughput fabrication of the devices without damaging the soft substrate (Figure 2). Moreover, the roughness and porosity of nitrocellulose allowed excellent adhesion and stability of the silver and PEDOT:PSS inks, eliminating the need for preparatory layers. After curing, the silver electrodes exhibited a resistance per unit length (3.71 ± 0.81 Ω/cm) comparable to that of silver tracks printed on a smooth Kapton substrate (2.8 ± 0.38 Ω/cm). The channel, gate and extended gate are arranged in a planar configuration and were fabricated by consecutive printing of three layers of PEDOT:PSS (Figure 2). We assessed the thickness *d* of the PEDOT:PSS penetrated in the paper via microscopy images of the device cross sections, which resulted to be approximately 5 μm (Figure 2b,c). In the same cross-sectional images, it can be observed that the PEDOT:PSS penetration depth is not uniform. This inhomogeneity may be attributed to local variations in cellulose absorption properties and pore distribution, resulting in uneven ink spreading throughout the substrate. Considering that film morphology can significantly impact device performance,^27^ variability in ink penetration might contribute to observed device-to-device differences. Introducing an insulating preparatory layer to fill pores before PEDOT:PSS printing could mitigate issues related to cellulose porosity. However, since this approach would compromise cellulose’s inherent microfluidic capabilities, it could only be applied in the dry area of the device, where liquid transport via capillarity is not essential. The surface morphology of bare nitrocellulose and printed PEDOT:PSS was evaluated via SEM imaging (Figure 2d–f). The bare substrate displays a fibrous structure with pore diameters of approximately 10 μm, allowing unhindered analytes transport (Figure 2d). In Figure 2e, the first PEDOT:PSS layer appears as a thin coating around the nitrocellulose fibers, while Figure 2f shows how successive printed layers form a more interconnected PEDOT:PSS matrix. To support this observations, we electrically characterized the printed films by measuring the resistance of one, two, and three layers of PEDOT:PSS, observing an increase in conductivity with the number of layers (Table S3). Transfer characteristics were then acquired, revealing that devices with three printed layers exhibited the highest transconductance (Figure S2), consistent with literature reports.^19,37^ The final number of layers was selected to achieve an optimal balance between transconductance and response speed, as higher transconductance is generally associated with slower device response times.^38^

**Figure 2:**
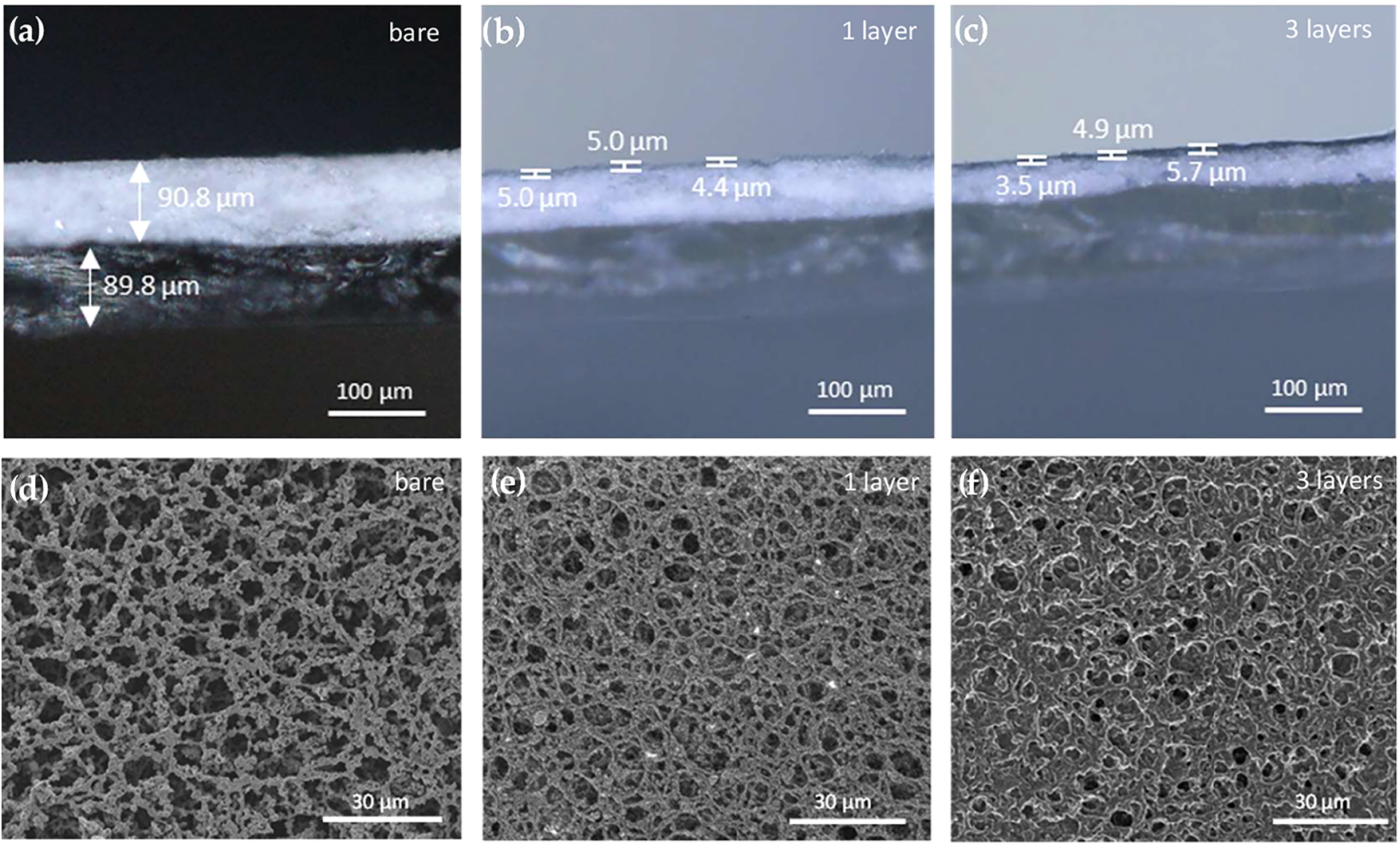
Top row: microscopy images of cross sections of (a) bare nitrocellulose, with its PET backing layer, (b) one layer and (c) three layers of PEDOT:PSS printed on nitrocellulose. The scale bar is 100 μm. Bottom row: SEM images of the surface of (d) nitrocellulose metallized by 50 nm of sputtered Platinum, (e) one layer and (f) three layers of PEDOT:PSS printed on nitrocellulose. The scale bar is 30 μm.

After printing the PEDOT:PSS components, the silver tracks were passivated to prevent contact with electrolytes and preserve their integrity. Al_2_O_3_ was chosen as a passivation material due to its compatibility with the substrate and for the high scalability of the e-beam evaporation process. A Kapton sheet was laser-cut to obtain a custom evaporation mask. The mask was aligned onto the printed devices to selectively deposit a 70 nm layer of Al_2_O_3_ on the electrodes.

Channel contamination in biosensing electrolyte-gated transistors has been traditionally addressed using microfluidic systems, which enable precise flow control through pump mechanisms.^39^ A potential advancement lies in the use of paper, which inherently serves as a self-standing microfluidic platform. With appropriate surface modifications, paper can be customized to achieve effective flow separation, offering a practical alternative to conventional PDMS-based systems. Since the discontinuation of solid ink printers, researchers have been seeking alternatives to wax printing for fabricating hydrophobic paths on paper, which until recently constituted a widely used and rapid method for producing cellulose-based microfluidic platforms.^40,41^ In this work, a common permanent marker was used to create a hydrophobic barrier separating the extended gate (EG) from the channel and gate (G) (Figure 1b) as a practical, low-cost solution aligned with our cost-effective approach. In the dry area enclosed in the drawn barriers, the communication between channel and gate was ensured by an SSE layer. This strategy preserves the channel from contamination and unspecific interaction with the analyte, keeping it isolated from the liquid sample soaking the rest of the nitrocellulose strip. Moreover, by locally applying and cross-linking the SSE only where needed, it was possible to minimize the interaction between the silver electrodes and the electrolyte, which could otherwise compromise the system response stability. The performance of the device was compared using 100 mM NaCl delivered through the nitrocellulose strip, in the presence or absence of the hydrophobic barrier and the SSE layer. The transfer characteristics under these two conditions reveal that the SSE layer dramatically improved the device’s stability compared to NaCl alone as the electrolyte (Figure 3a). This can be explained through the fact that the NaCl solution completely soaked the membrane, ultimately leading to oxidation of the nonpassivated silver at the contact pads. In contrast, localized application of the SSE prevented interaction between the electrolyte and the silver, demonstrating that the separation between wet and dry zones is crucial for the device’s reliability. To further asses electrical stability in the presence of the barriers and the SSE layer, we evaluated the transfer characteristics over 15 measurement cycles, observing an average drift per cycle of approximately 0.3% in *I*_on_ and 0.5% in *I*_off_ (Figure S1). After 15 cycles, *I*_on_ is 95.7% of *I*_on,0_ (the value of *I*_on_ at the first cycle), which is comparable with previous results on stability found in literature.^42,43^

**Figure 3:**
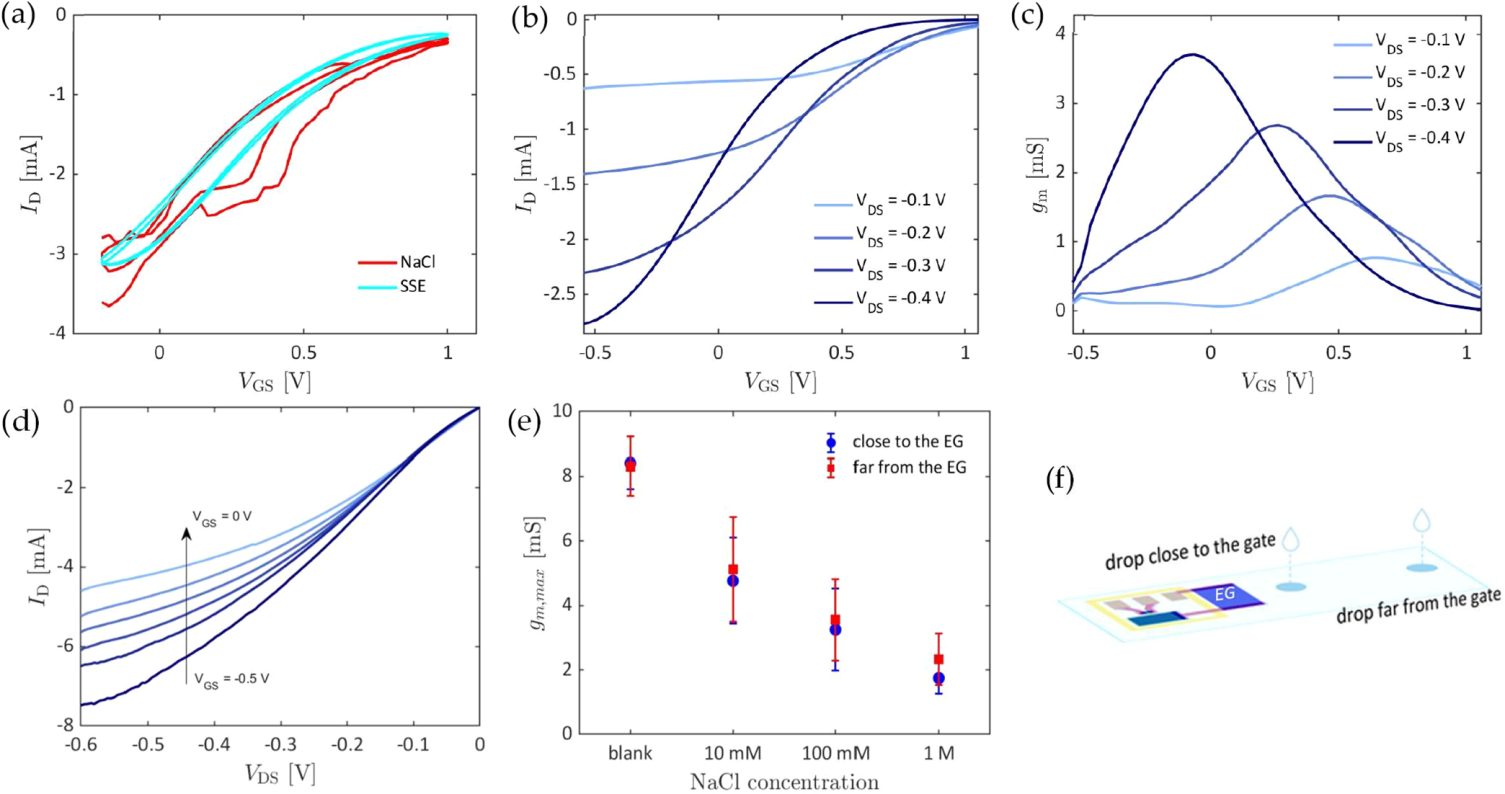
(a) Comparison between three cycles of transfer characteristic of a nitrocellulose-based OECT (*V*_GS_ = −0.4 V), using an SSE layer (red curves) or 100 mM NaCl solution (blue curves) as the electrolyte. (b) Transfer characteristic and (c) transconductance of a device at different drain–source voltages, using SSE as electrolyte on channel and gate and a drop of 100 mM NaCl solution on the extended gate. (d) Output characteristics of the same device at different gate voltages. (e) Comparison between averaged *g*_m,max_ of 3 devices, when dropping 50 μL of NaCl at different concentrations on the nitrocellulose strip, at 0.5 cm (close) or 1.5 cm (far) from the extended gate. (f) Schematic of the OECT printed on the nitrocellulose strip used to carry the liquid sample from different distances before the measure.

The environmental stability of the device components was assessed by measuring the resistance of the channel and the extended gate 6 weeks after fabrication, showing that both the silver tracks and the PEDOT:PSS remained stable under ambient conditions (Table S4). Similarly, the hand-drawn hydrophobic barriers preserved their functionality, effectively preventing liquid permeation in the dry area even after several weeks of storage (Figure S3). While the device components exhibit good stability under ambient conditions, the capillarity of cellulose slowly drains liquid from the SSE matrix in the dry area. Consequently, a few hours after SSE deposition, only an on-current can be measured, preventing the device from being switched off. Adding a preparatory layer able to fill the substrate pores in the dry area could be explored as a strategy to address this issue, so to enable long-term operation.

In the proposed wet-and-dry configuration, when a positive gate voltage is applied at the PEDOT:PSS extended gate in the wet zone, which is electrically connected to the gate in the dry zone, the channel OSC is reduced upon the injection of [EMIM]^+^ ions from the ionic liquid contained in the SSE layer,^35^ mediated by the presence of water in the compound. The transfer characteristics and output characteristics (Figure 3b,d) of the system were acquired at a scan rate of 61.2 mV/s, after dropping 50 μL of a 100 mM NaCl solution on the nitrocellulose strip before each measurement, allowing the flow to reach the extended gate. The transfer curves were used to extrapolate the transconductance *g*_m_, calculated as *g*_m_ = $\frac{dI_{D}}{dV_{\mathrm{GS}}}$ (Figure 3c). Transconductance, which reflects the signal amplification, is a key figure of merit for OECTs, and its maximum value, *g*_m,max_, is often reported to allow comparison between devices with uniform geometrical parameters. To account for the device-to-device variability, the maximum transconductance *g*_m,max_ was here normalized multiplying by the factor *d* * *W*/*L*, where *d* is the measured thickness of the penetrated film in the nitrocellulose, *W* is the width and *L* is the length of the channel. After compensating for the measured channel dimensions, the normalized *g*_m,max_ was determined to be 95,9 ± 13,3 S·nm. Another key figure of merit extracted from the transfer curves is the *I*_on_/*I*_off_ ratio, which is defined as the ratio between the maximum absolute value of the channel current *I*_D_ in the ON state and its minimum absolute value in the OFF state. Taking *I*_on_ = *I*_D_(V_GS_ = −0.6 V) and *I*_off_ = *I*_D_(V_GS_ = 1.2 V), the resulting *I*_on_/*I*_off_ ratio was found to be on the order of 10^2^, comparable with state-of-the-art devices.

To assess the system’s suitability for the integration in a LFA strip, where the sample travels by capillarity across the nitrocellulose membrane for about 1.5 cm before reaching the sensing lines, we investigated how the distance from the sample drop point to the extended gate affects the device response (Figure 3e,f). In Figure 3e, showing the averaged *g*_m,max_ of 3 devices for the two tested dropping points at different NaCl concentrations, it can be observed that the travel distance of the sample does not significantly impact on the device response, although a small dilution trend can be detected on the far-dropped samples.

To validate the performance of the OECT on paper, its response was analyzed by varying concentrations of dopamine (DA) in a 100 mM NaCl solution. Dopamine is a neurotransmitter produced by dopaminergic neurons in the central nervous system and can be found in human biological fluids—like cerebrospinal fluid and serum—in concentrations on the order of the nanomolar.^44^ Recent studies have linked altered dopamine levels to schizophrenia, Parkinson’s disease, and attention-deficit hyperactivity disorder (ADHD),^45–48^ driving interest toward the development of highly sensitive dopamine sensors. In addition to its biomedical relevance, dopamine has been utilized as a model analyte for validating OECT-based biosensors,^49–51^ owing to its sensitivity to applied voltage that results in its oxidation. In this work, 50 μL of dopamine solution at different concentrations, prepared in 100 mM NaCl, were dropped at the far end of the paper substrate. Once the sample reached the extended gate, the transfer characteristics were acquired at a scan rate of 61.2 mV/s. The applied potential at the extended gate caused the oxidation of dopamine to dopaquinone, producing 2 electrons and 2 protons (Figure 4a). The products of the reaction modify the electrical properties of the extended gate, which in turn alters the effective voltage dropping on the dry area of the system (Figure 4b). After a stabilization phase of approximately 5 min, corresponding to the first 6 cycles of measure, the response of the device to different DA concentration was investigated by extrapolating the *I*_on_/*I*_off_ ratio (Figure 4c) from the transfer characteristics (Figure 4b). Figure 4c shows how, starting from an average value of about 600 for the blank sample, *I*_on_/*I*_off_ decreases by increasing the DA concentration (*I*_on_ = *I*_D_(V_GS_ = −0.6 V) and *I*_off_ = *I*_D_(*V*_GS_ = 1.2 V)). This effect can be likely associated with the increasing dedoping of the PEDOT:PSS forming the extended gate and interacting with the products of DA oxidation. This configuration allows to isolate the effect of electrochemical events happening at sensing interface, avoiding interference of other active parts of the OECTs with the analyte. The maximum transconductance *g*_m,max_ was extrapolated from the transconductance curves to obtain the calibration plot for DA (Figure 4d,e). The limit of detection (LOD) was calculated via the 3σ method, where σ is the standard deviation of the blank measurements, finding a value of 0.01 mM. Like other cellulose-based lateral flow assays, our device is intended for single use, as analyte molecules remaining in the pores of the extended gate after sample permeation would prevent restoration of a pristine blank response.

**Figure 4:**
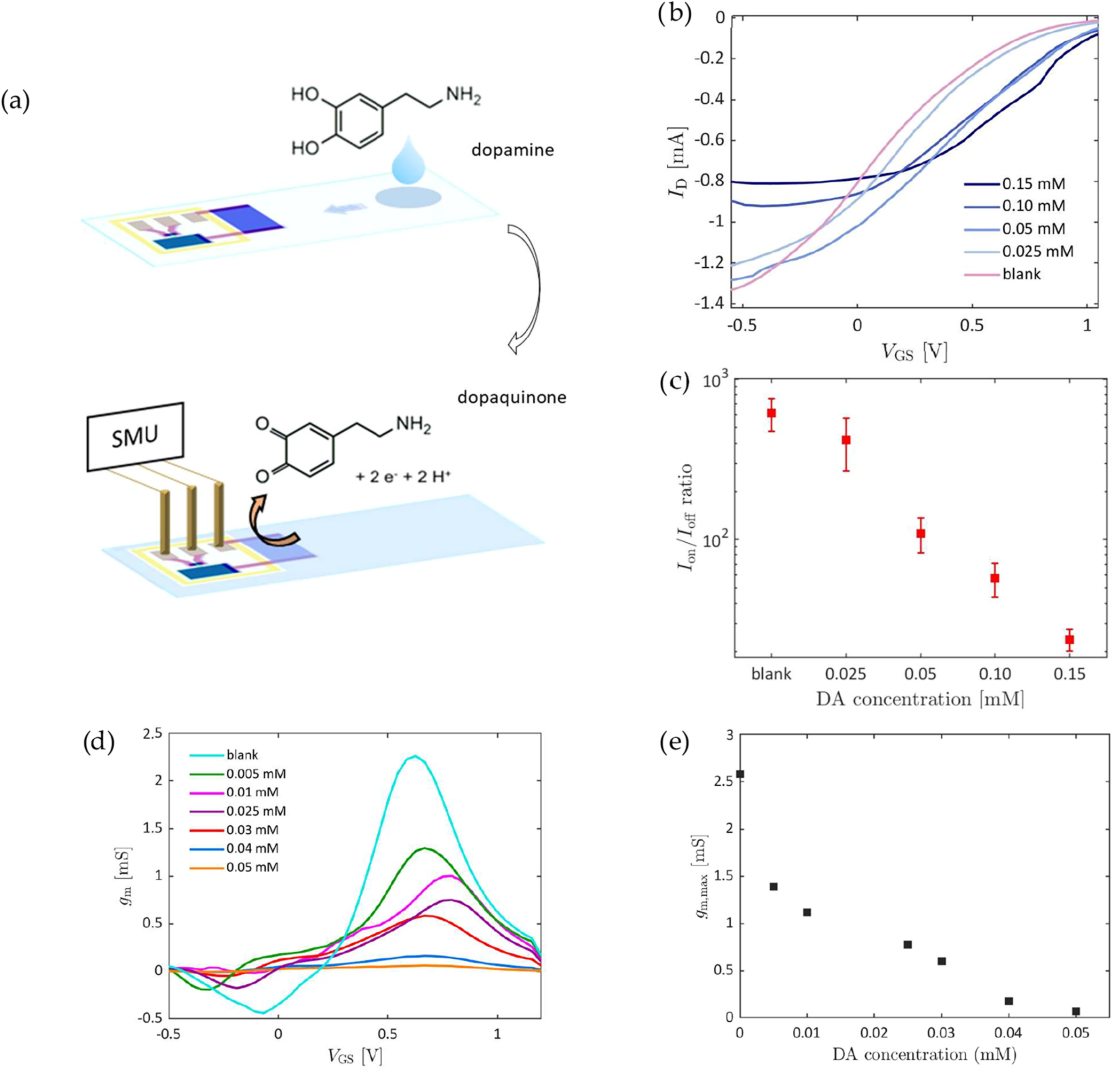
(a) Schematics of the sensing measurement steps. In the top image, 50 μL of DA solution are dropped on the far end of the nitrocellulose strip, allowing the flow to reach the extended gate. In the bottom image, a potential *V*_GS_ is applied to the extended gate, oxidizing the dopamine to dopaquinone. (b) Transfer characteristics of a device at different DA concentrations. (c) *I*_on_/*I*_off_ at different DA concentrations, averaged on 3 different devices. (d) Transconductance at different DA concentrations. (e) Calibration plot obtained for dopamine.

To evaluate device performance in the presence of an interferent for dopamine, we tested device response to increasing concentrations of uric acid (UA), finding that the gate potential *V*_g_ associated with the *g*_m,max_ values for UA tends to be higher compared to those associated with DA for concentrations higher than 0.025 mM (Figure S4), consistent with UA higher oxidation potential.^49,51^ However, when testing a solution containing both UA at the fixed concentration of 0.1 mM and increasing concentrations of DA, it was not possible to identify separate responses to the two molecules, making the device in its current configuration nonspecific for dopamine (Figure S4).

## Conclusions

This work presents a low-cost, paper-based OECT designed for integration into a single use lateral flow assays (LFAs). Following the principles that fuel the research behind point-of-care sensors, as cost-accessibility and lightweight, we focused on pursuing a low-cost fabrication route, estimated to yield a cost-per-device in the order of a few euros (Tables S1 and S2). The use of a cellulose substrate, along with a plastic-based gate and extended gate, and the silver ink were all chosen as contributing factors to reduce production costs while ensuring device quality and performances. Dispense printing using a commercial PCB printer was selected as a cost-effective, high-throughput additive manufacturing technique aimed at increasing device accessibility. While the passivation step was performed using a more traditional and cost-intensive method, the Al_2_O_3_ evaporation cost impact can be minimized through mass production, which optimizes the fixed costs associated with the process (Table S1). Together with the SSE role in enhancing device stability by minimizing silver-electrolyte interactions, the hand-drawn hydrophobic barrier enabled effective separation between wet and dry zones, a key feature for preserving channel stability over time. Although the functionality of the boundary was not affected by the variability in shape introduced by the hand-drawn hydrophobic barriers, this approach does pose limitations for scalability. This step could be automated through robotic systems capable of consistently drawing operator-independent lines using pens or markers. For large-scale production, adopting inkjet printing or screen-printing technologies could further optimize fabrication costs, enabling the patterning of barriers, insulating layers, and all other functional device components. Together with the device low operating voltage that minimizes power consumption, the easy readout of currents in the mA range makes system operation and signal analysis straightforward for potential application in portable platforms. Preliminary tests demonstrate robust device operation when integrated into a capillary-driven nitrocellulose strip, exhibiting a LOD of 0.01 mM for dopamine. Although the lowest detected concentration in this work is higher than the ideal sensitivity required for dopamine sensors, these results show promise for the easy integration of a OECT in a simple microfluidic system, paving the way for future coupling with nanoparticle-based labels that could enhance the system sensitivity. Additionally, the selectivity of the system could be addressed in future works through the functionalization of the extended gate, whose configuration offers high surface area for biorecognition molecules immobilization. This approach supports the development of widespread, point-of-care diagnostic devices, reinforcing the feasibility of scalable, affordable, and fully integrated biosensing platforms based on printed paper electronics.

## Supplementary Material



## Notes

Additional details regarding cost per device estimates; additional experimental details, including device electrical stability analysis, evidence on optimization of the number of layers of PEDOT:PSS, environmental stability and measurements with interferent (PDF)
