# Supplementary material for: A Printable OECT for Simple Integration in Nitrocellulose-Based Assays
Source: ACS Sens. 2025 Sep 30;10(10):7630–8. doi: 10.1021/acssensors.5c01893 (PMC12560127; doi:10.1021/acssensors.5c01893)
Supplement: Supplementary file 1 [file se5c01893_si_001.pdf]

Supporting information

A printable OECT for simple integration in nitrocellulose-based assays

Martina Cicolini[a,c], Ali Solgi[e], Lorenzo Vigna[a,c] *, Alberto Ballesio[a,c,d], Simone Marasso[a,c,d], Matteo Cocuzza[a,c], Hans Kleemann[e], Francesca Frascella[a,b], Lucia Napione[a,b]

a. Department of Applied Science and Technology (DISAT), Politecnico di Torino, Corso Duca Degli Abruzzi 24, 10129 Turin, Italy
b. PolitoBioMed Lab, Department of Applied Science and Technology (DISAT), Politecnico di Torino, Corso Duca Degli Abruzzi 24, 10129 Turin, Italy
c. PiQuET LAB, Piemonte Quantum Enabling Technology, Str. delle Cacce, 91, 10135 Turin, Italy
d. Institute of Materials for Electronics and Magnetism, IMEM-CNR, Parco Area delle Scienze 37/A, 43124 Parma, Italy
e. Dresden Integrated Center for Applied Physics and Photonic Materials (IAPP), Technische Universität Dresden, Nöthnitzer Str. 61, 01187 Dresden, Germany
* E-mail: lorenzo.vigna@polito.it;

Content

1. **Cost estimate per device**
2. **Electrical stability**
3. **Printing parameters optimization**
4. **Environmental stability**
5. **Interferent measurement**

1. Cost estimate per device

Table S1. Overview of the estimated costs per device.

Equipment	Supplier price (€)	Estimate cost per device (€)
Nitrocellulose	131 for 10 sheets[1]	0.08
Silver ink	~ 90 for 2 ml[2]	0.09
PEDOT:PSS ink	~ 300 for 100 g[3]	0.02
Printing nozzle	22 for 25 nozzles[4]	0.01
Permanent marker	1.5 per marker	0.03
SSE	12.14 for 2.5 ml	0.015
Kapton mask	~ 70 for 5 sheets[5]	0.60
Al_2O_3 layer	~ 65 per run	0.25
	**Total cost per device**	~ 1.10

In table S1, an overview of the estimated costs per device is reported. A detailed breakdown of each entry can be provided as follows:

- Nitrocellulose: considering a sample area of 1x1.5 cm^2, plus some offset to easily cut the strips in separate samples, we can estimate that an A4 sheet of nitrocellulose can accommodate about 178 samples with an area of 3.5 cm^2. With a cost of 131 euros per 10 sheets, we can approximate the cost per device concerning the substrate to 0.08 euros.
- Silver ink: considering that the sum of the areas of the silver elements constituting one device is about 17 mm^2, and having observed that the thickness of a silver track printed on a silicon substrate is about 3 µm, we can estimate the volume of silver ink used for one device to be around 0.051 mm^3. Considering the loss of ink that occurs when calibrating the ink flux before printing and when changing a nozzle, and therefore

estimating an ink consumption of about 2 ml per 1000 devices, we can approximate the cost per device concerning the silver ink consumption to 0.09 euros.

- PEDOT:PSS ink: considering a higher ink loss during flux calibration due to lower density, estimating an ink use of about 3 g per 500 devices, we can approximate the cost per device concerning the PEDOT:PSS ink consumption to 0.02 euros.
- Printing nozzles: assuming a change of nozzle after printing 200 devices, and considering the use of 2 different nozzles for each device – one dedicated to the silver ink, the other dedicated to the PEDOT:PSS ink – , we can estimate the cost per device concerning the consumption of nozzles to be approximately 0.01 euros.
- Permanent marker: assuming to be able to realize 50 devices with one marker before experiencing a reduction in ink flow, we can estimate a cost per device concerning the realization of the hydrophobic barriers of 0.03 euros.
- SSE: considering a cost per microliter of 0.005 euros, we can estimate a cost per device concerning the SSE layer of 0.015 euros circa.

Table S2. Overview of the estimated costs to prepare 2.5 ml of SSE.

**Ingredient**	**Amount used for 2.5 ml batch of SSE**	**Supplier price (€)**	**Unit price**	**Cost for 2.5 ml batch of SSE**
N-isopropylacrylamide	750.0 mg	~ 60 per 10 g[6]	0.006/ mg	4.50 €
Crosslinker	20.0 mg	~ 60 per 100 g[7]	0.0006/ mg	0.01 €
Photo-initiator	200.0 mg	~ 100 per 5 g[8]	0.02/ mg	4.00 €
Ionic liquid	1.5 mL (~2.015 g)*	~ 180 per 100 g[9]	1.80/ g	3.63 €
DI Water	1 ml		negligible	

*Density of [EMIM][$EtSO_4$] ≈ 1.343 g/ml → 1.5 ml × 1.343 g/ml ≈ 2.015 g

Total cost of a 2.5 mL batch ≈ 12.14 €

12.14 € ÷ 2500 µL ≈ 0.005 €/µl

Cost for 3 µl (amount used per device) = 0.015 €

- Laser-cut Kapton mask: for the Kapton masks, we account for a cost per package of Kapton sheets of 70 euros, where a package contains 5 A4 sheets of 25 micrometers thickness, and each sheet can accommodate around 6 mask of 10 cm. Each mask can host approximately 20 devices. Estimating a cost of 5 euros for the laser cutter use and usure, and 5 euros for power, we can approximate a total cost per mask concerning the laser cutting process to 10 euros. Considering a cost per mask of 2.33 euros for materials and 10 euros for fabrication, we can estimate a cost per device concerning the realization of the mask passivation layer of 0.60 euros circa.
- Al_2O_3 evaporation: each of the 13 wafers of 4 inches (10.16 cm) hosted in the rotating dome of the e-beam evaporator used in this work can be adopted as support to host approximately 20 devices. Estimating a cost of 30 euros of material, 30 euros for facility use and usure, and 5 euros for power, we can approximate a total cost per run of 65 euros. Assuming to fill the dome with 260 samples (20 samples per wafer), we can estimate a cost per device concerning the realization of the passivation layer of 0.25 euros circa.
- Voltera V-One: the cost of the Voltera V-One PCB printer is around 3500 euros[10]. With a printing area of 148.5 cm^2, each printing run allows to print approximately 42 samples, taking around 30 seconds per device.

As a reference, the price of commercially available screen-printed electrochemical sensors ranges from a few euros to approximately 20 euros, depending on the materials and specifications [11,12].

2. Electrical stability

Transfer characteristics were acquired to evaluate the device stability over 15 cycles of measurements, after soaking the extended gate in 100 mM NaCl. After a stabilization period consisting of the first five cycles, an average drift per cycle of approximately 0.3% in I_{on} and 0.5% in I_{off} was observed.

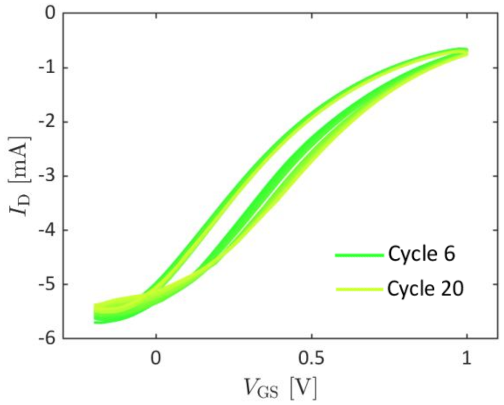

Figure S1. Device transfer characteristics over 15 cycles of measure.

3. Printing parameters optimization

The resistivity of PEDOT:PSS films was measured to determine the optimal number of printed layers. As shown in Table S3, conductivity increases with the number of layers, in agreement with previously reported results in the literature[13,14]. Similarly, the transconductance of the fabricated OECTs is highest for devices prepared with three printed PEDOT:PSS layers (Figure S2).

Table S3. Resistance values of 1 layer, 2 layers and 3 layers of printed PEDOT:PSS channel and extended gate.

	1 layer	2 layers	3 layers
Channel	257 ± 83 Ω	82 ± 11 Ω	29 ± 3 Ω
Extended gate	10 ± 2 kΩ	6.6 ± 2.8 kΩ	0.9 ± 0.2 kΩ

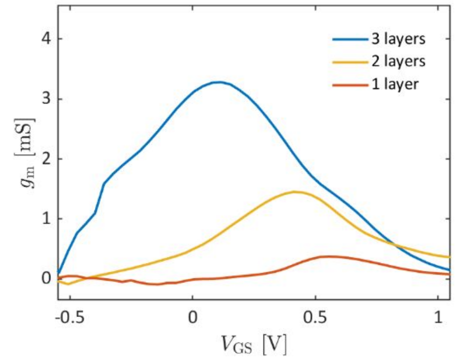

Figure S2. Transconductance of devices with 1 layer, 2 layers and 3 layers of printed PEDOT:PSS channel, gate, and extended gate.

4. Environmental stability

Table S4. Resistance values for 3 layers of printed PEDOT:PSS channel and extended gate over time.

**3 layers**	t=0	t=2 weeks	t=6 weeks
Channel	29 ± 3 Ω	31 ± 4 Ω	35 ± 6 Ω
Extended gate	0.9 ± 0.2 kΩ	0.9 ± 0.2 kΩ	1 ± 0.1 kΩ

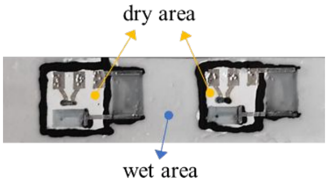

Figure S3. Picture of two printed devices, taken 6 weeks after fabrication. The nitrocellulose substrate was soaked with water to assess the stability and function of the hydrophobic barriers, which had been drawn with a black permanent marker immediately after printing.

5. Interferent measurement

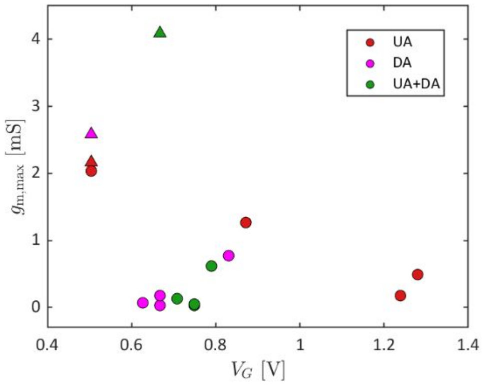

Figure S4. Maximum transconductance of devices tested with UA, DA, and UA+DA solutions. UA and DA were tested at 0.025, 0.05, 0.1, and 0.15 mM. For UA+DA solutions, UA was fixed at 0.1 mM while DA was varied (0.025, 0.05, 0.1, and 0.15 mM). Triangles represent the blank measure for each test.

References

(1) Whatman® FF170HP Din A Nitrocellulose sheets. https://www.sigmaaldrich.com/IT/it/product/aldrich/wha13549204?srsltid=AfmBOorUr6lwn7vL5sdU0RzMBAX-zKV2-gnKiEkCAAgI2s_njdBW3l_t.

(2) Voltera Conductor 3 - Silver Ink. https://store.voltera.io/products/conductor-3-ink-cartridge-2ml.

(3) S V4 PEDOT:PSS. https://www.ossila.com/products/pedot-pss-s-v4-stab.

(4) Voltera Disposable Nozzles. https://store.voltera.io/products/nozzles-disposable.

(5) Kapton HN Goodfellow Sheets. Https://It.Rs-Online.Com/Web/p/Pellicole-in-Plastica/5363946?Gb=s.

(6) N-Isopropylacrylamide. https://www.sigmaaldrich.com/IT/it/product/aldrich/415324.

(7) N,N′-Methylenebis(acrylamide). https://www.sigmaaldrich.com/IT/it/product/sial/146072.

(8) Photoinitiator. https://www.cellink.com/product/irgacure-2959-photoinitiator/?n=1.

(9) 1-Ethyl-3-methylimidazolium ethyl sulfate. https://www.sigmaaldrich.com/IT/it/product/aldrich/51682.

(10) Voltera V-One. https://store.voltera.io/products/v-one?variant=45963756306654.

(11) Screen printed electrodes. https://shop.zimmerpeacock.com/products/hyper-value-501-carbon-electrode?variant=40553384345674.

(12) Screen printed electrodes. https://www.sigmaaldrich.com/IT/it/search/screen-printed-electrode?focus=products&page=1&perpage=30&sort=relevance&term=screen%20printed%20electrode&type=product.

(13) Ait Yazza, A.; Blondeau, P.; Andrade, F. J. Simple Approach for Building High Transconductance Paper-Based Organic Electrochemical Transistor (OECT) for Chemical Sensing. ACS Appl Electron Mater **2021**, 3 (4), 1886–1895. https://doi.org/10.1021/acsaelm.1c00116.

(14) Rivnay, J.; Inal, S.; Salleo, A.; Owens, R. M.; Berggren, M.; Malliaras, G. G. Organic Electrochemical Transistors. Nature Reviews Materials. Nature Publishing Group January 16, 2018. https://doi.org/10.1038/natrevmats.2017.86.
